# Effect of Dietary Organic Selenium on Growth Performance, Gut Health, and Coccidiosis Response in Broiler Chickens

**DOI:** 10.3390/ani13091560

**Published:** 2023-05-06

**Authors:** Samiru S. Wickramasuriya, Inkyung Park, Youngsub Lee, Hyun S. Lillehoj

**Affiliations:** Animal Bioscience and Biotechnology Laboratory, United States Department of Agriculture, Agricultural Research Service, Beltsville, MD 20705, USA; samiru.sudharaka@usda.gov (S.S.W.);

**Keywords:** selenium, broiler, coccidiosis, antibiotic alternative, gut health, oxidative stress

## Abstract

**Simple Summary:**

Selenium is an essential trace mineral for better performance, meat quality, and health benefits in farm animals. In broiler diets, selenium has been added in inorganic or organic form to maintain the selenium requirement of the chickens. Researchers have studied the effects and applications of both inorganic and organic selenium in broiler nutrition. However, there is a paucity of information pertaining to organic selenium application under coccidiosis conditions. In this study, we evaluated the effect of dietary organic selenium on growth performance, gut health, and tissue selenium concentrations in broiler chickens under coccidiosis conditions. Results indicated that the addition of organic selenized yeast improved the growth performance and enhanced the selenium concentrations in the tissue, regardless of the *Eimeria* challenge. Therefore, organic selenized yeast supplementation can be recommended to produce selenium-enriched organic broiler chickens.

**Abstract:**

A total of 252 one-day-old Ross broilers were randomly allocated to one of six treatments in a 2 × 3 factorial arrangement with respective *Eimeria* challenges (non-infection and infection) and three different selenium (Se) diets. Dietary treatments were as follows: (1) Se un-supplemented control (CON), (2) inorganic Se treatment (SS; 0.3 mg/kg as sodium selenite), and (3) organic Se treatment (SY; 0.3 mg/kg as selenized yeast). Six replicate cages were allocated per treatment. Chickens in the respective *Eimeria* infection groups were infected with an *E. acervulina*, *E. tenella*, and *E. maxima* oocyst mixture (15,000 oocysts/chicken) on day 16. Growth performance was measured on days 16, 22, and 24. On day 22, intestinal samples were collected from randomly selected chickens to evaluate gut lesion scores, antioxidant enzymes, and tight junction gene expression. Blood, breast, and liver samples were collected to analyze the Se concentrations on day 24. Dietary SY supplementation improved (*p* < 0.05) the growth performance of the chickens regardless of the *Eimeria* challenge. Moreover, independent of *Eimeria* infection, Se supplementation elevated (*p* < 0.05) the heme oxygenase 1 (HMOX-1) expression in jejunal mucosa at 6 days post-infection (dpi). Duodenal junctional adhesion molecule 2 (JAM-2) expression and jejunal occludin (OCLN) were elevated (*p* < 0.05) with dietary SY supplementation at 6 dpi. Among Se sources, broiler chickens fed with the SY diet showed higher (*p* < 0.05) Se concentrations in breast muscle and serum on 8 dpi. These results confirmed the beneficial effects of dietary Se and the efficiency of organic Se compared with inorganic Se for growth improvement and muscle Se enrichment in broiler chickens regardless of coccidiosis infection.

## 1. Introduction

Trace mineral selenium (Se) has been used in poultry diets for a few decades because of its well-known health benefits. Some of these benefits include maintaining the growth performance, redox potential, reproduction, and immune function of the chickens [1]. In broiler diets, Se is added in inorganic or organic form to maintain the Se requirement of the chickens. Conventionally, sodium selenite (Na_2_SeO_3_) and sodium selenate (Na_2_SeO_4_) are the most widely used Se sources in broiler diet formulations in the form of inorganic Se. However, organic sources of Se, such as seleno-methionine (C_5_H_11_NO_2_Se), seleno-cysteine (C_3_H_7_NO_2_Se), and Se-enriched yeast, have become popular and many broiler experiments have been conducted due to their higher bioavailability and tissue retention compared to inorganic Se [1]. In addition, a new form of mineral nanoelemental Se was also developed, tested, and showed improved performance in broiler chickens [2].

The impact of dietary Se supplementation on broiler chickens varies to a great extent with different factors, such as the environment, Se source, and Se dosage levels. In recent years, broiler chickens were used to evaluate the multifactorial interaction of Se nutrition with the growth performance, immunity, gut health, and antioxidant potentials. The impacts of Se and Se sources on heat stress, high stocking density, and disease challenge conditions in broiler chickens were reported [3,4]. According to Sun et al. [3], organic Se facilitated better performance and immune status in broiler chickens under high stocking density and heat stress challenges. Similarly, the importance of organic Se in mitigating inflammation and oxidative stress under heat stress conditions was observed with commercial layer chickens [5]. Moreover, it was reported that broiler chickens fed organic Se (as Se yeast) had better oxidative stress resistance during *Escherichia coli* challenge and heat stress conditions [6]. In another study, dietary Se enhanced chicken immunity during the vaccine response to the low-pathogenicity avian influenza virus [7].

As an enteric infection, coccidiosis is a major broiler disease condition caused by *Eimeria* protozoan parasites, resulting in poor nutrient absorption and hindering growth performance via gut epithelial damage [8,9,10]. In recent papers, the annual loss due to coccidiosis was reported to be over USD 14 billion for the poultry industry globally [10,11]. Before the regulatory antibiotic ban in the livestock industry, enteric infection conditions were successfully managed with in-feed antibiotics. Afterward, different dietary antibiotic alternative feed additives were tested, including probiotics, prebiotics [12], organic acids, enzymes [13,14], phytochemicals [15], and amino acids [16], to mitigate the coccidiosis in broiler chickens [10]. Moreover, dietary vitamins and/or trace minerals’ effects on coccidiosis control were documented [17]. El-Maddawy et al. [18] reported the beneficial effects of zinc oxide nanoparticles as anticoccidial agents in broiler chickens. Similarly, the influence of dietary zinc, copper, and manganese on the performance and intestinal health of coccidiosis-afflicted broiler chickens was reported [19]. Previously, we reported the beneficial effect of organic B-Traxim Se in protecting young broiler chickens against necrotic enteritis [20,21,22].

In an early study [23], protective immunity against *Eimeria tenella* was reported with inorganic Se. Nevertheless, the response to dietary Se supplementation and organic Se sources during coccidiosis infection conditions in broiler chickens is scantly documented. With the mounting concern regarding organic poultry products in the market, it is important to understand the broiler responses to dietary organic Se and its tissue accumulation dynamics. The objective of the present study was to understand the effect of dietary organic Se supplementation on performance, gut health, and tissue Se concentrations in coccidiosis-infected broiler chickens.

## 2. Materials and Methods

### 2.1. Chickens and Animal Care

Day-old male broiler chicks (Ross 708) were transferred from a local hatchery (Longnecker Hatchery, Elizabethtown, PA, USA) to the experimental farm. Upon arrival, a total of 252 healthy chicks were weighed individually and randomly allocated to six treatments in a 2 × 3 factorial treatment arrangement, maintaining the same body weight (44.03 ± 0.2 g) and weight distribution among treatments and replicates. Each treatment contained six replicate cages with seven chickens each. Up to 14 days of age, chickens were raised in electrically heated Petersime brooder units housed in a temperature-controlled closed-house environment. After 14 days, chickens were moved to experimental grower cages. All the management practices followed the Ross broiler management guide.

### 2.2. Experimental Design, Diets, and Treatments

**Figure 1:**
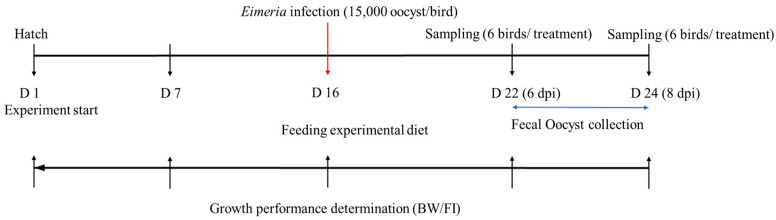
Schematic outline of the experimental design.

The experiment was performed using a completely randomized design in a factorial arrangement with respective factors being the three different Se diets and the two different *Eimeria* challenge conditions (non-infection and infection). Dietary treatments included (1) Se un-supplemented control (CON), (2) inorganic Se-supplemented treatment (SS; 0.3 mg/kg as sodium selenite), and (3) organic Se-supplemented treatment (SY; 0.3 mg/kg as selenized yeast, Sel-Plex^®^, Alltech, Nicholasville, KY, USA). All experimental diets were formulated and manufactured (NC State University feed mill, Raleigh, NC, USA) based on corn and soybean meal to meet or exceed the nutritional requirements and Se was supplemented accordingly (Table 1). Chickens were fed with the non-medicated respective treatment diets until the end of the experimental period. *Ad libitum* feed and fresh clean water were provided at all times. To obtain the two different *Eimeria* challenge conditions, chickens were infected with a freshly propagated and sporulated oocyst mixture (15,000 oocyst/mL/chicken; 5000/mL *E. acervulina*, 5000/mL *E. tenella*, and 5000/mL *E. maxima*) through oral gavage, except for the chickens in the uninfected treatments, on day 16 (Figure 1). In order to make the sporulated oocyst mixture, laboratory-maintained and genotypically verified pure *Eimeria* strains were mixed proportionately after enumeration. Uninfected chickens received phosphate-buffered saline (PBS) as a placebo in place of the sporulated oocyst mixture.

### 2.3. Growth Performance Evaluation

All chickens were weighed on days 16, 22, and 24, and we recorded the pen basis body weights to calculate the average daily gain (ADG). Feed disappearance of the individual cages was recorded to measure the average daily feed intake (ADFI). The feed conversion ratio (FCR) was calculated and corrected for mortalities.

### 2.4. Sample Collection

On day 22 (6 days post-infection (dpi)), six chickens from each treatment group (1 bird from each replicate) were randomly selected for sample collection, considering this as the peak infection time. After sacrificing chickens by manual cervical dislocation, 15-cm-long mid-duodenal and jejunal samples together with whole ceca were obtained for gut lesion scoring. The remaining duodenum and jejunum tissue samples were scraped aseptically to collect the mucosa using a tissue scraper [9] for antioxidant enzyme and tight junction gene expression analysis. Collected mucosa samples were stored in RNA stabilization solution (RNAlater™ solution, Invitrogen Corporation, Carlsbad, CA, USA) at −20 °C.

Blood samples were collected on day 24 (8 dpi) for serum separation (5 chickens/treatment) followed by cervical dislocation. Separated sera from collected blood samples were stored at −20 °C to analyze the Se content. At the same time, breast muscle samples (pectoralis major and minor) and liver samples were collected from the same chickens to analyze the Se concentration.

### 2.5. Gut Lesion Scoring

Gut samples were evaluated by three independent scientists based on the gut lesion scoring technique, as described previously [24]. Briefly, intestinal samples and ceca were placed on a white background in good light and slit open. Both the unopened serosal area and the opened mucosal surface were examined for lesions. A four-point hedonic scale was used to enumerate the lesion severity.

### 2.6. Se Analysis

Collected serum, breast muscle, and liver samples were frozen (−20 °C) and sent to the Michigan State University Veterinary Diagnostic Laboratory (Veterinary Diagnostic Laboratory, Lansing, MI, USA) for tissue Se analysis. After tissue preparation, the Se content of the samples was analyzed using an Agilent 7900 inductively coupled plasma–mass spectrometer (Agilent Technologies Inc., Santa Clara, CA, USA), as described previously [25]. In brief, an aliquot of each diluted tissue digest and calibration standard was diluted 25-fold with a solution containing 0.5% ethylenediaminetetraacetic acid and Triton X-100, 1% ammonium hydroxide, 2% butanol, and 5ppb of scandium and 7.5 ppb of germanium, rhodium, indium, and bismuth as internal standards. The ICP/MS was tuned to yield a minimum of 7500 cps sensitivity for 1 ppb yttrium (mass 89), less than a 1.0% oxide level as determined by the 156/140 mass ratio, and less than 2.0% double charged ions as determined by the 70/140 mass ratio. Elemental concentrations were calibrated using a 6-point linear curve of the analyte–internal standard response ratio. Standards were from Inorganic Ventures (Inorganic Ventures, Christainsburg, VA, USA). Bovine liver and mussel standards (National Institute of Standards and Technology, Gaithersburg, MD, USA) were used as controls. A second source calibration check standard from Alfa Aesar (Alfa Aesar, Tewksbury, MA, USA) was also used.

### 2.7. Oocyst Counts

From 6 dpi (day 22) to 8 dpi (day 24), fecal samples from every cage were collected separately to enumerate the oocyst shedding, considering this period as the peak oocyst production period. The collected fecal samples were processed according to the method described previously [26]. Briefly, total feces collected from individual cages were soaked and homogenized with 3 L of water. Two subsamples from each cage were separated into 50 mL tubes for oocyst counting. Subsamples were then subjected to serial dilutions before the enumeration of oocysts for each sample. Three individual scientists counted oocysts microscopically with a McMaster counting chamber (Challex LLC, Park City, UT, USA) using the sodium chloride flotation method [26]. The total number of oocysts shed per chicken was calculated using the following formula:

Total oocysts = (oocyst count × dilution factor × fecal sample volume/counting chamber volume)/number of chickens per cage.

### 2.8. Real-Time PCR Analysis

Total RNA from each tissue sample was extracted and homogenized using TRIzol reagent (Invitrogen, Carlsbad, CA, USA), followed by DNase digestion, as described [12]. The RNA quantity and purity were assessed using a NanoDrop spectrophotometer (NanoDrop One; Thermo Scientific, Wilmington, DE, USA) at 260/280 nm. Extracted RNA was diluted to the same concentration and the synthesis of cDNA was performed using a QuantiTect^®^ Reverse Transcription Kit (Qiagen, Hilden, Germany), as per the manufacturer’s instructions. The cDNA samples were diluted to 1:5, and 5 µL aliquots were used for qRT-PCR amplification. Each sample was analyzed using SYBR Green qPCR Master Mix (PowerTrack, Applied Biosystems, Vilnius, Lithuania) in triplicate, using the Applied Biosystems QuantStudio 3 Real-Time PCR Systems (Life Technologies, Carlsbad, CA, USA). The following PCR conditions were followed: denaturation at 95 °C for 2 min followed by 40 cycles of 95 °C for 15 s and 60 °C for 1 min s. Glyceraldehyde 3-phosphate dehydrogenase (GAPDH) was used as the housekeeping gene for gene expression. For the relative quantification of the gene expression levels, the logarithmic-scaled threshold cycle (Ct) values were used in the 2^−∆∆Ct^ method before calculating the mean and standard error of the mean (SEM) for the references and individual targets.

The encoded gene expression levels of tight junction proteins, including junctional adhesion molecule 2 (JAM-2), occludin (OCLN), and zonula occludens 1 (ZO-1), mucin (MUC-2) expression in the intestinal samples, and antioxidant gene expression, such as superoxide dismutase type 1 (SOD-1), catalase (CAT), and heme oxygenase 1 (HMOX-1), were investigated. All oligonucleotide sequences of the forward and reverse primers used in this experiment are listed in Table 2.

### 2.9. Statistical Analysis

Data were analyzed as a completely randomized design, using a general linear model procedure of two-way ANOVA in the SPSS software (Version 24; IBM SPSS 2016, Armonk, NY, USA). *Eimeria* infection and dietary Se were considered as the two main effects. The pen was used as the experimental unit for all growth performance measurements. The selected individual chicken was considered as the replicate unit for other measurements. Mean differences were considered significant at *p* < 0.05. When treatment effects were significant (*p* < 0.05), means were separated using Duncan multiple range test procedures.

## 3. Results

### 3.1. Growth Performance

Dietary Se or *Eimeria* infection did not show any interaction effect (*p* > 0.05) on the growth performance of broiler chickens from hatching to 24 days of age (Table 3). Regardless of *Eimeria* infection, dietary Se supplementation improved (*p* < 0.05) the ADG of broiler chickens from hatching to 22 days of age. Interestingly, chickens fed SY diets showed a higher ADG from the beginning of the experiment up to day 22. However, SS’ impact (*p* < 0.05) on ADG was shown after the *Eimeria* infection (Day 16). Se supplementation did not affect (*p* > 0.05) the ADFI of broiler chickens from day 1 to day 16. Afterward, from day 16 to 22, SS and SY diet-fed chickens showed a lower feed intake (*p* < 0.05) followed by better feed efficiency (*p* < 0.05) compared to CON chickens. Regardless of dietary treatment, *Eimeria* infection reduced (*p* < 0.05) the ADG and ADFI of broiler chickens from day 16 to day 24. Similarly, the FCR was increased (*p* < 0.05) in the *Eimeria*-infected broiler chickens from day 16 to day 22.

### 3.2. Fecal Oocyst Shedding

**Figure 2:**
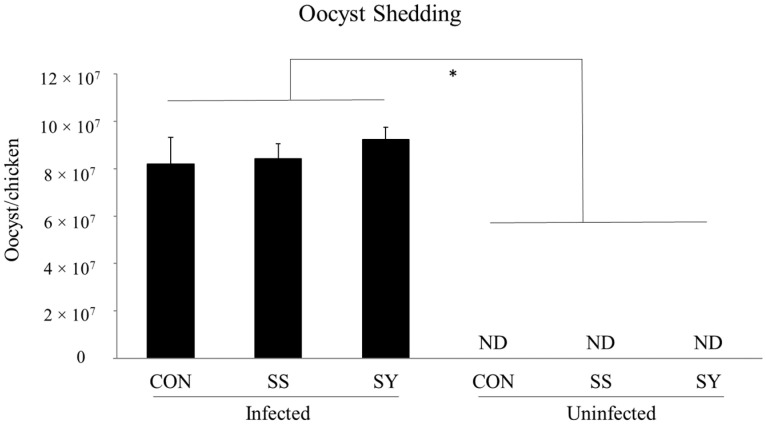
Effect of selenium supplementation on oocyst shedding of mixed *Eimeria* (*E. tenella*, *E. acervulina*, and *E. maxima*) infected broiler chickens. Chickens were infected (15,000 oocyst/chicken) with freshly propagated and sporulated oocyst mixture with 5000/mL *E. acervulina*, 5000/mL *E. tenella*, and 5000/mL *E. maxima* via oral gavage on day 16. CON: Se-un-supplemented diet, SS: inorganic Se-supplemented diet (0.3 mg/kg as sodium selenite), SY: organic selenized yeast-supplemented diet (0.3 mg/kg, Sel-Plex^®^, Alltech), ND: not detected. Each bar represents the mean ± SEM (*n* = 6). * indicates significant (*p* < 0.05) difference. Fecal samples were collected from 6 to 8 days post-infection to calculate the oocyst shedding.

Dietary Se and *Eimeria* infection did not show any interaction effect (*p* > 0.05) on fecal oocyst shedding. Moreover, dietary Se supplementation (or Se source) did not affect (*p* > 0.05) the fecal oocyst shedding in *Eimeria*-infected broiler chickens (Figure 2). Regardless of dietary Se, *Eimeria* infection significantly increased (*p* < 0.05) the fecal oocyst shedding from 6 to 8 dpi.

### 3.3. Intestinal Lesion Scores

**Figure 3:**
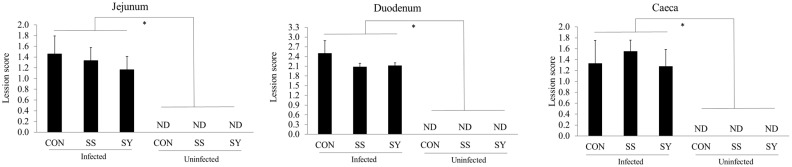
Effect of selenium supplementation on intestinal lesion scores of mixed *Eimeria*-infected broiler chickens. Chickens were infected (15,000 oocyst/chicken) with freshly propagated and sporulated oocyst mixture with 5000/mL *E. acervulina*, 5000/mL *E. tenella*, and 5000/mL *E. maxima* through oral gavage on day 16. CON: Se-un-supplemented diet, SS: inorganic Se-supplemented diet (0.3 mg/kg as sodium selenite), SY: organic selenized yeast-supplemented diet (0.3 mg/kg, Sel-Plex^®^, Alltech), ND: not detected. Each bar represents the mean ± SEM (*n* = 6). * indicates significant (*p* < 0.05) difference. Intestinal tissue samples were collected from 6 days post-infection to assess intestinal lesion score.

There were no interaction effects (*p* > 0.05) between dietary Se and *Eimeria* infection for the intestinal lesion scores of broiler chickens at day 22 (6 dpi; Figure 3). For the main effect of *Eimeria* infection, broiler chickens infected with *Eimeria* showed increased (*p* < 0.05) gut lesion scores on day 24 (8 dpi), independent of Se treatments. However, dietary Se did not affect (*p* > 0.05) the intestinal lesion scores of broiler chickens, although Se supplementation lowered the jejunal and duodenal lesion scores numerically.

### 3.4. Antioxidant Gene Expression

The effects of dietary Se and *Eimeria* infection on the mucosal antioxidant gene expression of broiler chickens are presented in Table 4. There were no interaction effects (*p* > 0.05) between dietary Se and *Eimeria* infection on the duodenum and jejunum antioxidant gene expression at 6 dpi. Moreover, as the main effect, dietary Se or *Eimeria* infection did not change (*p* > 0.05) the SOD-1 and CAT gene expression levels in the duodenum and jejunum mucosa. The main effect was that the *Eimeria* infection lowered (*p* < 0.05) the HMOX-1 gene expression in the duodenum mucosa compared to the uninfected chickens. Regardless of *Eimeria* infection, Se supplementation (either SS or SY) elevated (*p* < 0.05) the HMOX-1 expression in the jejunum mucosa.

### 3.5. Tight Junction and Mucin Gene Expression

There were no interaction effects (*p* > 0.05) between dietary Se and *Eimeria* infection on the duodenum and jejunum tight junction gene expression of broiler chickens at day 22 (6 dpi; Table 5). Regardless of *Eimeria* infection, SY supplementation elevated (*p* < 0.05) the JAM-2 expression in the duodenum mucosa of broiler chickens compared to the SS counterpart. Similarly, SY supplementation elevated (*p* < 0.05) the OCLN expression in the jejunum mucosa of the broiler chickens. Independent of dietary treatments, *Eimeria* infection reduced (*p* < 0.05) the OCLN and MUC-2 gene expression in the jejunum mucosa.

### 3.6. Tissue Se Concentration

**Figure 4:**
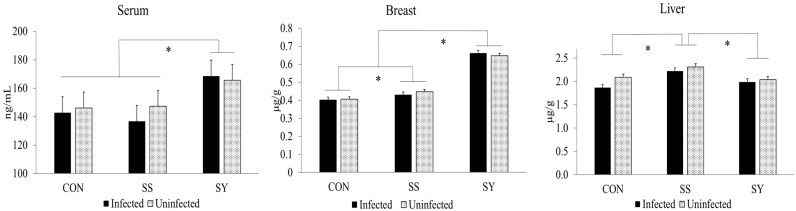
Effect of dietary Se and *Eimeria* infection on selenium concentrations in serum, breast, and liver tissue of broiler chickens. * indicates significant (*p* < 0.05) difference. Chickens were infected (15,000 oocyst/chicken) with freshly propagated and sporulated oocyst mixture with 5000/mL *E. acervulina*, 5000/mL *E. tenella*, and 5000/mL *E. maxima* through oral gavage on day 16. CON: Se-un-supplemented diet, SS: inorganic Se-supplemented diet (0.3 mg/kg as sodium selenite), and SY: organic selenized yeast-supplemented diet (0.3 mg/kg, Sel-Plex^®^, Alltech). Each bar represents the mean ± SEM (*n* = 6). Tissue samples were collected at 8 days post-infection.

No interaction effects (*p* > 0.05) were observed between dietary Se and *Eimeria* infection on Se concentrations in the tissue of broiler chickens at day 24 (8 dpi; Figure 4). Regardless of *Eimeria* infection, broiler chickens fed the SY diet showed elevated (*p* < 0.05) Se content in the serum and breast muscle compared to chickens fed other treatment diets on day 24. However, SS-fed chickens showed higher (*p* < 0.05) Se retention in liver tissue compared to CON- and SY-fed chickens.

## 4. Discussion

The chicken responses to dietary Se supplementation and different Se sources have been reported under various stress and challenge conditions [3,4,27]. However, there is a lack of knowledge and understanding of broiler responses to dietary organic Se supplementation following coccidiosis infection. In this regard, the present study was conducted to investigate the effect of dietary inorganic and organic Se in broiler diets on growth performance, oocyst shedding, gut health, and tissue Se concentrations in coccidiosis-infected broiler chickens.

In this study, Se supplementation and *Eimeria* infection did not show any significant interaction effects on the growth performance of broiler chickens. Regardless of Se supplementation, mixed *Eimeria* infection reduced the growth performance of broiler chickens, similarly to previous studies [9,28]. As the main effect, the addition of Se into the diet (both organic and inorganic) improved the growth performance of broiler chickens in this study, which was identical to previous studies that reported the beneficial effects of dietary Se on chicken growth responses [22]. Dietary Se works as an activator and cofactor of key enzyme 5′ deiodinase for triiodo thryonine synthesis, where growth is controlled by the energy and protein assimilation of the animal [29]. Thus, it is postulated that Se supplementation improves the growth performance of chickens through improved protein and energy digestibility. Nevertheless, some other studies did not show significant growth differences in broiler chickens with dietary Se supplementation [30]. The disparity between these different observations may be explained by the initial Se content of the basal diets in the experiments. When the initial Se content in the diet is lower than the requirement for chickens, broiler chickens positively respond to dietary Se supplementation, and therefore studies conducted with sufficient Se in the control diet did not show any growth response in chickens [30]. In this study, we maintained 0.35 mg/kg Se in our Se-supplemented diets and 0.07 mg/kg in the Se-un-supplemented control diets. In our control diet, the Se level was lower than the National Research Council (1994) recommendation (0.1 mg/kg) for optimum growth in broilers. Looking into the dietary Se sources, previous researchers [30,31,32,33] reported no growth performance difference in chickens fed inorganic and organic Se. However, the results of this study showed an improved body weight and ADG in SY-fed broiler chickens compared to the SS-fed chickens up to day 16 of age. Similar to our findings, Upton et al. [34] and Sundu et al. [35] observed improved growth performance when broiler chickens were fed with an organic selenized yeast (Sel-plex)-supplemented diet. The inconsistency of these growth results with other studies may be caused by many factors, including the dietary Se content in basal diets, chicken breed, age, and experimental conditions. The mechanisms by which organic Se improves the growth performance of broiler chickens depend upon higher bioavailability, improved tight junction permeability, and reduced oxidative stress in young broiler chickens [22,36]. Moreover, it is worth noting that the chickens in the CON group showed higher feed intake compared to the chickens in other treatments from days 16 to 22. It is possible that the lower dietary Se may have increased the feed intake and thereby lowered the feed efficiency in broiler chickens. The observed feed intake results agree with a previous report [37] that showed that broiler chickens fed 0.1 mg/kg consumed more feed compared to those fed 0.25 mg/kg Se. The reason for the higher feed intake in low-Se-fed broiler chickens may be due to feather growth and the metabolism of thyroid hormones. As explained by Choct et al. [37], lower dietary Se concentrations reduce feather growth, leading to higher maintenance energy requirements in chickens. Consequently, chickens tend to eat more feed to maintain the energy requirement.

To understand the effects of dietary Se supplementation and the source of Se on *Eimeria* oocyst shedding, we enumerated the fecal oocysts in this study. *Eimeria*-infected broiler chickens showed significantly higher oocyst shedding regardless of dietary Se, as we observed in our previous studies [9,28,38]. According to our observation, neither Se supplementation nor the Se source affected the fecal oocyst shedding of the broiler chickens. However, Mengistu et al. [39] reported lower oocyst shedding in *E. tenella*-infected chickens fed with sodium selenite-supplemented diets. Similarly, a previous study in mice [40] observed lower fecal oocyst shedding of *E. papillate* with a Se-supplemented diet. The possible mechanism of *Eimeria* inhibition by dietary Se could be explained as a direct inhibitory effect on *Eimeria*, or indirect suppression via local oxidative burst, altered microbiota, and the blocking of intracellular parasite development [40]. The disparity observed between our study and these previous studies may be explained by the different animal models used, the dose of Se, and/or different *Eimeria* species with different virulency.

When *Eimeria* penetrates host intestinal epithelial cells to complete its life cycle, it causes visible gross lesions, and it can be used as a tool to assess the severity of coccidiosis infections [12]. Based on the site specificity of the *Eimeria* spp., we measured the intestinal lesions in the duodenum, jejunum, and caeca, as we used an *Eimeria* oocyst mixture that contained *E. tenella*, *E. acervulina*, and *E. maxima*. Broiler chickens infected with *Eimeria* showed higher gut lesions compared to the uninfected chickens in all three measured sites, regardless of dietary treatments. The invasion of *Eimeria* sporozoites and merozoites into intestinal epithelial cells and their intracellular replication led to local inflammation in the intestinal mucosa, accompanied by the infiltration of various types of leukocytes [41,42,43]. Therefore, our observation is in agreement with the previous literature that has described elevated gut lesions in *Eimeria*-infected chickens [38,44]. In this study, dietary Se supplementation did not significantly affect the intestinal lesion scores, regardless of *Eimeria* infection. However, although statistically not significant, jejunum and duodenum lesion scores were numerically lower in chickens fed with Se-supplemented diets compared to those of chickens without Se-supplemented diets. Similar to our results, Georgieva et al. [45] also failed to see a significant sodium selenite impact on the gut lesions of *E. acervulina*-infected chickens. Nooreh et al. [44] reported that a combination of vitamins E, C, and Se lowered the intestinal lesion scores of chickens infected with mixed *Eimeria* parasites to conclude that the beneficial effect may have been driven by the vitamins and not the Se. In another study, Mengistu et al. [39] observed lower gut lesion scores in *E. tenella*-infected chickens fed sodium selenite-supplemented diets compared to Se-un-supplemented control chickens. The reason that there was no significant difference in gut lesion scores could be due to the higher virulency of the *Eimeria* spp. that we used to challenge in this study.

In modern poultry production, genetic selection for rapid growth, higher feed efficiency, and egg production in a confined cage system promotes high oxidative stress conditions, especially in newly hatched chickens [46,47]. In addition, in newly hatched chickens, until they develop adaptive immunity, the occurrence of enteric diseases such as coccidiosis and necrotic enteritis contributes to high levels of oxidative stress, leading to lower antioxidant gene expression in broiler chickens, especially when their maternal immunity starts to wane at around three weeks [9,48]. In this study, we observed lower HMOX-1 gene expression in the duodenal mucosa in *Eimeria*-infected chickens regardless of dietary treatments. HMOX-1 is a rate-limiting enzyme responsible for catalyzing the reaction that degrades heme to biliverdin, and we observed lower HMOX-1 gene expression in the mucosa and spleen in *E. acervulina*-infected broiler chickens in our previous study [9], similar to the present study. As an essential element for the antioxidant enzyme system, Se is vital for detoxifying lipid peroxide and reactive oxygen species, which are generated from the oxidative stress response [49]. Although antioxidant gene expression patterns in the mucosa were not reflective of the Se responses, as we expected, elevated HMOX-1 gene expression was observed in the jejunal mucosa regardless of the *Eimeria* challenge, showing the dietary Se effect on the mucosal antioxidant system. In a recent study [50], dietary supplementation of organic Se increased the serum T-AOC activity but not the levels of GSH-Px, SOD, or MDA activity. Similarly, we did not observe a significant difference in SOD-1 and CAT activity in the duodenum and jejunum mucosa of the Se-supplemented diet-fed broiler chickens in this study. However, previous studies [2,3] reported the positive effect of Se in terms of enhanced antioxidant defense systems in broiler chickens under different stress conditions. The disparity between these and our results may be due to fewer stress responses in our birds, as observed in the SOD-1 and CAT activity.

To further understand the impact and interactions of dietary Se and *Eimeria* infection on broiler gut health, we assessed the tight junction and mucin gene expression in the duodenal and jejunal mucosa. The tight junction is an integral part of the intercellular junctional complex of intestinal epithelial cells that plays a vital role to maintain the paracellular permeability and thereby the gut health of the host animal [28]. In this regard, the comparative expression levels of the tight junctional genes such as JAM-2, OCLN, and ZO-1 were used as biomarkers to assess the gut health of the broiler chickens [42]. The results of the present study showed that chickens challenged with *Eimeria* demonstrated lower expression of OCLN and MUC-2 genes in the jejunum. Similar to the present results, previous studies from our laboratory [28,42] reported downregulated tight junction gene expression with *Eimeria* infection, indicating the detrimental effect of intracellular parasitism on the gut health of young broiler chickens. Moreover, our previous study also showed that the mucin (MUC-2) gene expression, which is a key encoded secretory protein for gut barrier protection, was downregulated when chickens were infected with coccidiosis [9]. As the main effect, chickens fed with an organic selenized yeast-supplemented diet showed upregulated JAM-2 and OCLN gene expression compared to their inorganic Se-fed counterparts in the duodenum and jejunum, respectively. These results support the notion that organic selenized yeast promotes chicken gut health by enhancing structural gut integrity and by decreasing intestinal epithelial permeability. According to Yang et al. [51], Se-enriched yeast inhibited the NF-κB signaling pathway and reduced the intestinal tight junction injury caused by ochratoxin A toxin in broilers. Likewise, the results of this study showing the upregulated tight junction gene expression support the important role of organic selenized yeast in improving gut health.

According to our data, breast and liver tissue showed a higher Se concentration compared to the serum in all treatment groups, and it is not surprising that body tissue such as the liver accumulates more Se regardless of the Se source or the dietary level [52]. However, the bioavailability of each Se source is a key factor that governs the Se concentrations in host animal tissue. Organic selenized yeast has been reported to contain predominantly selenomethionine (50–70%) and more than 100 other unique Se species [27]. Therefore, selenized yeast shown to provide higher bioavailability compared to inorganic sodium selenite to promote higher Se concentrations in broiler chickens [3,29]. In agreement, organic selenized yeast showed a higher Se concentration in serum and breast meat [52,53]. Nevertheless, sodium selenite-fed chickens showed elevated Se retention in the liver compared to organic selenized yeast-fed chickens. Similar to our results, higher selenium retention in the liver was previously reported by Ibrahim et al. [54]. Presumably, the underlying reason for the higher level of Se in the liver may be caused by the absorption pathways, with lesser bioaccumulation in body muscles. In agreement with this idea, previous reports [29,36,55] confirmed that organic selenized yeast metabolizes and is then absorbed as selenomethionine and selenocysteine via an active mechanism, similar to methionine (neutral amino acid transport system), and deposition occurs in the muscle tissue as a source of methionine instead of Se and thereby lowers the Se concentrations in the liver. With regard to *Eimeria* infection, the tissue Se concentration was not affected by the infection in this study. Moreover, the reduced stress conditions affirmed by the antioxidant gene expression as discussed above may explain the lack of a significant Se concentration difference in the tissue between *Eimeria*-infected and uninfected chickens.

## 5. Conclusions

In conclusion, the present study on the role of dietary Se supplementation in coccidiosis-infected young broiler chickens showed that dietary Se supplementation did not interact with coccidiosis conditions in growth performance, intestinal health, oocyst shedding, and tissue Se concentrations. Regardless of the *Eimeria* challenge, Se supplementation in broiler diets improved the growth performance of the broiler chickens. Specifically, the addition of organic selenized yeast improved the growth performance and enhanced the Se concentrations in the tissue; therefore, organic Se supplementation is beneficial to produce Se-enriched organic broiler chickens.

## Figures and Tables

**Table 1:** Nutrition composition (% as-fed basis) of the experimental diets ^1^.

Ingredients	CON	SS	SY
Corn	63.36	63.45	63.38
Soybean Meal	31.69	31.68	31.68
Soybean Oil	1.51	1.48	1.51
Defluorinated Phosphate	1.12	1.12	1.12
Limestone	0.51	0.46	0.50
DL-Methionine	0.38	0.38	0.38
L-Lysine, HCl	0.35	0.35	0.35
Salt	0.30	0.30	0.30
Sodium Carbonate	0.20	0.20	0.20
L-Threonine	0.17	0.17	0.17
L-Valine	0.07	0.07	0.07
Choline Chloride	0.05	0.05	0.05
Mineral Mix ^2^	0.16	0.16	0.16
Vitamin Premix ^3^	0.06	0.06	0.06
Phytase, 5000 FTU/g	0.02	0.02	0.02
Sand	0.05	0.00	0.00
Sodium Selenite	0.00	0.05	0.00
Seleno Yeast	0.00	0.00	0.05
Calculated Nutrients			
AME, kcal/kg	3045	3045	3045
Crude Protein, %	20.61	20.61	20.61
Dig. Lysine, %	1.20	1.20	1.20
Dig. Threonine, %	0.82	0.82	0.82
Dig. Methionine, %	0.66	0.66	0.66
Dig. Cysteine, %	0.26	0.26	0.26
Calcium, %	0.90	0.90	0.90
Total Phosphorus, %	0.55	0.55	0.55
Available Phosphorus, %	0.45	0.45	0.45
Analyzed Value			
Selenium, mg/kg	0.07	0.36	0.35

^1^ CON: Se-un-supplemented diet, SS: inorganic Se-supplemented diet (0.3 mg/kg as sodium selenite), and SY: organic selenized yeast-supplemented diet (0.3 mg/kg, Sel-Plex^®^, Alltech). ^2^ Mineral premix supplied the following per kilogram of diet: 120 mg manganese, 120 mg zinc, 80 mg iron, 10 mg copper, 2.5 mg iodine, and 1 mg cobalt. ^3^ Vitamin premix supplied the following per kilogram of diet: 13,200 IU vitamin A, 4000 IU vitamin D3, 33 IU vitamin E, 0.02 mg vitamin B12, 0.13 mg biotin, 2 mg menadione (K3), 2 mg thiamine, 6.6 mg riboflavin, 11 mg d-pantothenic acid, 4 mg vitamin B6, 55 mg niacin, and 1.1 mg folic acid.

**Table 2:** Quantitative real-time PCR oligonucleotide primer sequences ^1^.

Target Gene	Primer Sequence	Accession No.
GAPDH	F: 5′-GGTGGTGCTAAGCGTGTTAT-3′	K01458
	R: 5′-ACCTCTGTCATCTCTCCACA-3′	
JAM-2	F: 5′-AGCCTCAAATGGGATTGGATT-3′	NM0,010,06257.1
	R: 5′-CATCAACTTGCATTCGCTTCA-3′	
OCLN	F: 5′-GAGCCCAGACTACCAAAGCAA-3′	NM205,128.1
	R: 5′-GCTTGATGTGGAAGAGCTTGTTG-3′	
ZO-1	F: 5′-CCGCAGTCGTTCACGATCT-3′	XM01,527,8981.1
	R: 5′-GGAGAATGTCTGGAATGGTCTGA-3′	
MUC-2	F: 5′-GCCTGCCCAGGAAATCAAG-3′	NM0,013,18434.1
	R: 5′-CGACAAGTTTGCTGGCACAT-3′	
HMOX-1	F: 5′-CTGGAGAAGGGTTGGCTTTCT-3′	NM205344
	R: 5′-GAAGCTCTGCCTTTGGCTGTA-3′	
SOD-1	F: 5′-ATTACCGGCTTGTCTGATGG-3′	NM205064.1
	R: 5′-CCTCCCTTTGCAGTCACATT-3′	
CAT	F: 5′-ACTGCAAGGCGAAAGTGTTT-3′	NM001031215.1
	R: 5′-GGCTATGGATGAAGGATGGA-3′	

^1^ Abbreviations: GAPDH, glyceraldehyde 3-phosphate dehydrogenase; JAM-2, junctional adhesion molecule 2; OCLN, occludin; ZO-1, zonula occludens 1; MUC-2, mucin 2; HMOX-1, heme oxygenase 1; SOD-1, superoxide dismutase 1; CAT, catalase; F, forward primer; R, reverse primer.

**Table 3:** Effect of dietary selenium source and *Eimeria* infection on growth performance of broiler chickens ^1^.

Item	Diet ^2^			SEM	Infection ^3^		SEM	*p*-Value		
	CON	SS	SY		Un-Infected	Infected		Diet	Infection	D × I
Body Weight, g										
Day 16	689.44 ^a^	695.44 ^a^	717.80 ^b^	7.672	700.16	701.62	6.265	0.034	0.871	0.348
Day 22 (6 dpi)	1112.91 ^a^	1128.89 ^ab^	1152.82 ^b^	12.670	1160.67	1102.41	10.345	0.092	0.001	0.834
Day 24 (8 dpi)	1322.43	1303.94	1323.28	16.272	1349.92	1283.19	13.904	0.645	0.001	0.206
Average Daily Gain, g/bird/day										
Day 1–16	38.53 ^a^	38.88 ^a^	40.22 ^b^	0.436	39.21	39.21	0.356	0.025	0.987	0.369
Day 16–22	67.42 ^a^	75.01 ^b^	72.90 ^b^	1.655	75.83	67.72	1.351	0.008	0.001	0.335
Day 16–24	75.76	80.64	79.15	2.229	82.02	75.02	1.905	0.335	0.013	0.120
Average Daily Feed Intake, g/bird/day										
Day 1–16	33.15	33.13	34.74	5.978	34.11	33.23	4.881	0.976	0.899	0.995
Day 16–22	115.61 ^b^	109.45 ^a^	112.74 ^a,b^	1.645	115.22	109.98	1.343	0.038	0.008	0.233
Day 16–24	125.66	119.24	122.96	2.094	125.96	119.28	1.709	0.104	0.008	0.216
Feed Conversion Rate, g/g										
Day 1–16	1.24	1.23	1.24	0.021	1.25	1.22	0.017	0.902	0.224	0.975
Day 16–22	1.66 ^b^	1.48 ^a^	1.53 ^a^	0.035	1.49	1.62	0.029	0.002	0.006	0.841
Day 16–24	1.63	1.50	1.58	0.049	1.54	1.60	0.040	0.169	0.301	0.929

^1^ SEM; pooled standard error of the mean. ^a,b^ Values in a row with different superscripts differ significantly (*p* < 0.05). ^2^ Values are the mean of 12 replicates per treatment. CON: Se-un-supplemented diet, SS: inorganic Se-supplemented diet (0.3 mg/kg as sodium selenite), and SY: organic selenized yeast-supplemented diet (0.3 mg/kg, Sel-Plex^®^, Alltech). ^3^ Values are the mean of 18 replicates per treatment; chickens were infected by oocysts of mixed *Eimeria* (*E. tenella*, *E. acervulina*, and *E. maxima*) using oral gavage at day 16 with 15,000 oocysts/chicken.

**Table 4:** Effect of dietary selenium and *Eimeria* infection on antioxidant gene expression of broiler chickens ^1^.

Item	Diet ^2^			SEM	Infection ^3^		SEM	*p*-Value		
	CON	SS	SY		Un-Infected	Infected		Diet	Infection	D × I
Duodenum										
SOD-1	3.02	1.89	1.72	0.468	2.43	1.99	0.382	0.119	0.420	0.767
CAT	0.53	0.38	0.38	0.077	0.41	0.45	0.063	0.289	0.618	0.316
HMOX-1	0.06	0.08	0.07	0.010	0.08	0.06	0.009	0.342	0.030	0.078
Jejunum										
SOD-1	2.95	4.85	5.51	1.257	3.55	5.32	0.954	0.306	0.193	0.073
CAT	0.27	0.26	0.36	0.049	0.31	0.29	0.041	0.352	0.745	0.963
HMOX-1	0.04 ^a^	0.07 ^b^	0.08 ^b^	0.009	0.07	0.06	0.007	0.040	0.331	0.778

^1^ HMOX-1, heme oxygenase 1; SOD-1, superoxide dismutase 1; CAT, catalase. SEM; pooled standard error of the mean. ^a,b^ Values in a row with different superscripts differ significantly (*p* < 0.05). ^2^ Values are the mean of 12 replicates per treatment. CON: Se-un-supplemented diet, SS: inorganic Se-supplemented diet (0.3 mg/kg as sodium selenite), and SY: organic selenized yeast-supplemented diet (0.3 mg/kg, Sel-Plex^®^, Alltech). ^3^ Values are the mean of 18 replicates per treatment; chickens were infected by oocysts of mixed *Eimeria* (*E. tenella*, *E. acervulina*, and *E. maxima*) using oral gavage on day 16 with 15,000 oocysts/chicken.

**Table 5:** Effect of dietary selenium source and *Eimeria* infection on the tight junction and mucin gene expression of broiler chickens ^1^.

Item	Diet ^2^			SEM	Infection ^3^		SEM	*p*-Value		
	CON	SS	SY		Un-Infected	Infected		Diet	Infection	D × I
Duodenum										
JAM-2	0.072 ^ab^	0.013 ^a^	0.15 ^b^	0.036	0.034	0.123	0.029	0.037	0.058	0.084
OCLN	0.164	0.097	0.187	0.046	0.114	0.185	0.038	0.367	0.197	0.398
ZO-1	0.207	0.099	0.341	0.073	0.151	0.281	0.062	0.085	0.136	0.136
MUC-2	1.396	0.846	1.392	0.374	1.04	1.382	0.322	0.526	0.446	0.256
Jejunum										
JAM-2	0.048	0.048	0.048	0.017	0.047	0.046	0.014	0.835	0.936	0.083
OCLN	0.078 ^a^	0.078 ^a^	0.129 ^b^	0.013	0.111	0.079	0.01	0.008	0.033	0.137
ZO-1	0.146	0.116	0.170	0.031	0.138	0.151	0.026	0.464	0.718	0.795
MUC-2	4.465	4.270	4.955	0.979	6.205	2.922	0.799	0.879	0.008	0.960

^1^ Transcript levels of junctional adhesion molecule 2 (JAM2), occludin (OCLN), zonula occludens 1 (ZO1), and mucin 2 (MUC-2) in duodenal and jejunal mucosa were measured by quantitative RT-PCR and gene expression was analyzed using the 2^−∆∆Ct^ method. SEM; pooled standard error of the mean. ^a,b^ Values in a row with different superscripts differ significantly (*p* < 0.05). ^2^ Values are the mean of 12 replicates per treatment: CON: Se-un-supplemented diet, SS: inorganic Se-supplemented diet (0.3 mg/kg as sodium selenite), and SY: organic selenized yeast-supplemented diet (0.3 mg/kg, Sel-Plex^®^, Alltech). ^3^ Values are the mean of 18 replicates per treatment; chickens were infected by oocysts of mixed *Eimeria* (*E. tenella*, *E. acervulina*, and *E. maxima*) using oral gavage on day 16 with 15,000 oocysts/chicken.

## Data Availability

The data presented in this study are available on request from the corresponding author.
